# Development and characterization of a novel nanobody with SRMV neutralizing activity

**DOI:** 10.1186/s12934-024-02311-6

**Published:** 2024-02-10

**Authors:** Miao Sun, Changjiang Wang, Huaye Luo, Yanfei Chen, Guanggang Qu, Jian Chen, Ling Li, Min Zhang, Qinghong Xue

**Affiliations:** 1 Department of Viral Biologics, China Institute of Veterinary Drug Control, Beijing, China; 2 Shandong Binzhou Animal Science and Veterinary Medicine Academy, Binzhou, China; 3 Shanghai Veterinary Research Institute, Chinese Academy of Agricultural Science, Shanghai, China; 4 Tech-Bank Food Corporation Limited, Nanjing, China

**Keywords:** Small ruminant morbillivirus (SRMV), Nanobody, Fusion protein, Hemagglutinin protein

## Abstract

Peste des petits ruminants (PPR) is an acute, contact infectious disease caused by the small ruminant morbillivirus (SRMV), and its morbidity in goats and sheep can be up to 100% with significant mortality. Nanobody generated from camelid animals such as alpaca has attracted wide attention because of its unique advantages compared with conventional antibodies. The main objective of this study was to produce specific nanobodies against SRMV and identify its characteristics. To obtain the coding gene of SRMV-specific nanobodies, we first constructed an immune phage-displayed library from the VHH repertoire of alpaca that was immunized with SRMV-F and -H proteins. By using phage display technology, the target antigen-specific VHHs can be obtained after four consecutive rounds of biopanning. Results showed that the size of this VHH library was 2.26 × 10^10^ CFU/mL and the SRMV-F and -H specific phage particles were greatly enriched after four rounds of biopanning. The positive phage clones were selected and sequenced, and total of five independent different sequences of SRMV-specific nanobodies were identified. Subsequently, the DNA fragments of the five nanobodies were cloned into *E. coli* BL21(DE3), respectively, and three of them were successfully expressed and purified. Specificity and affinity towards inactivated SRMV of these purified nanobodies were then evaluated using the ELISA method. Results demonstrated that NbSRMV-1-1, NbSRMV-2-10, and NbSRMV-1-21 showed no cross-reactivity with other antigens, such as inactivated BTV, inactivated FMDV, His-tag labeled protein, and BSA. The ELISA titer of these three nanobodies against inactivated SRMV was up to 1:1000. However, only NbSRMV-1-21 displayed SRMV neutralizing activity at a maximum dilution of 1:4. The results indicate that the nanobodies against SRMV generated in this study could be useful in future applications. This study provided a novel antibody tool and laid a foundation for the treatment and detection of SRMV.

## Introduction

Peste des petits ruminants (PPR) is a highly contagious and economically important ruminant disease that is caused by small ruminant morbillivirus (SRMV). It significantly impacts on the international movement and trade of sheep and goats, as well as their products, making it a major socio-economic concern worldwide [1, 2]. PPR is listed as an emerging disease under the Office International des Epizooties. In 2015, the United Nations Food and Agriculture Organization (FAO) and the World Organization for Animal Health (OIE) jointly established a global control and eradication strategy for PPR.

SRMV belongs to the genus *Morbillivirus* in the family *Paramyxoviridae* [3]. The genome of SRMV is a negative-strand unsegmented ssRNA [4], with a total length of about 16,000 bp, which encodes six structural proteins and two non-structural proteins (C and V) in the order 3′-N-P(C/V)M-F-H-L-5′ [5]. The surface glycoproteins, namely the haemagglutinin (H) and fusion (F) proteins, have been shown to confer protective immunity against SRMV [6–8]. Capripox recombinants expressing the H protein or the F protein of RPV conferred protection against PPR disease in goats [9].

The single-domain antibody (sdAb), comprising the variable region of its heavy chain, is commonly referred to as the variable domain of heavy chain antibodies (VHHs) or heavy chain antibodies (HcAb), also known as nanobodies (Nbs) [10]. VHHs have many advantages over conventional antibodies due to their small size, with a relative molecular mass of 15 kDa [11]. This compact size enables VHHs to effortlessly access target surfaces, including cracks and hidden epitopes [12, 13], and be easily bioengineered into novel bivalent/multivalent/multispecific and high-affinity molecules [14, 15]. The recombinant expression of VHHs in microbial systems and direct purification using His-tag by immobilized metal affinity chromatography makes purification easy and inexpensive [11, 16–18].

VHHs are versatile molecules with favorable properties and have been evaluated for immune-diagnosis, immune therapy, and immune analysis [10, 14, 19]. For example, VHHs have been used successfully to distinguish between Brucella and Yersinia infections in livestock, while traditional monoclonal antibodies (mAbs) have failed [20]. In recently, Wrapp et al. identify neutralizing cross-reactive single-domain camelid antibodies, which may serve as useful reagents for researchers studying the viruses causing MERS, SARS, and COVID-19 [21–23]. Other nanobodies also show broad application prospects in biochemistry, structural biology, and diagnostic assay development.

In this study, we successfully constructed the VHH phage display libraries by using RNA from peripheral blood lymphocytes of alpaca immunized with purified SRMV-F and -H proteins mixture. The VHHs against SRMV F and H separately were screened, and expressed by *Ecoli* system. After purification, the affinity and neutralizing activity of all VHHs were evaluated. Here, we generated SRMV-reactive nanobodies, which may provide an application foundation for the development of diagnostic and therapeutic neutralizing antibodies in the future.

## Materials and methods

### Antigens and antibodies

SRMV-F and -H proteins (full length) were prepared by the China Institute of Veterinary Drug Control by using CHO cells. HRP-rabbit anti-Llama IgG (Invitrogen, San Diego, CA, USA) and HRP-conjugated goat anti-rabbit IgG (Sigma-Aldrich, St. Louis, MO, USA) were purchased from Thermo Fisher Scientific Inc. HRP-conjugate anti-M13 monoclonal antibody was purchased from Sino Biological Inc. Primers used in this study was shown in Table 1.

**Table 1:** Primers used in this study

Primers	Sequence 5’-3’	Note
VHH1-F	GTCCTGGCTGCTCTTCTACAAGG	For library construction, first round of PCR reaction
VHH1-R	GGTACGTGCTGTTGAACTGTTCC	
VHH2-F	CTGGCCCAGCCGGCCATGGCTCAGTTGCAGCTCGTG	For library construction, second round of PCR reaction
VHH2-R	AAGGAAAAAAGCGGCCGCTGAGGAAACGGTGACCTGGG	
Detc-F	CCATGATTACGCCAAGCTTTGGAGCC	For positive rate identification of the library
Detc-R	CGATCTAAAGTTTTGTCGTCTTTCC	
Exp-F	CGCGGATCCATGGCTCAGGTGCAGCTCA	For expression of nanobodies against SRMV
Exp-R	CCCAAGCTTTGGTTGTGGTTTTGGTGTCTTGGG	

### Immunization of the alpaca and serum antibody titer identification

One adult male alpaca was immunized subcutaneously in the neck with purified SRMV-F (500 µg) and purified SRMV -H protein (500 µg) in an equal volume of Freund’s complete adjuvant, and after two weeks, five subsequent boosts were done with of the SRMV-F (200 µg) and SRMV-H protein (200 µg) emulsified in Freund’s incomplete adjuvant by biweekly intervals. Blood was obtained before the first injection, and one week after the sixth injection. Serum samples were tested for SRMV-specific antibody response by ELISA. Peripheral blood mononuclear cells (PBMCs) were isolated from the blood by using FICOLL (Solarbio, Beijing, China).

### Construction of VHH phage display library

Total mRNA was isolated from PBMCs, using Trizol according to the instructions (Invitrogen). cDNAs were amplified by using the SMART^TM^ RACE cDNA Amplification kit (Clontech) and Oligo(dT)20 primer. The variable region gene of the VHH was amplified by two rounds of PCR. The first PCR reaction was to obtain the length of approximately 700 bp fragment by using the primer pair VHH1-F and VHH1-R. The 700 bp fragment was purified using the QIAquick gel extraction kit (Qiagen) and was then used as a template in the second PCR reaction to obtain the length of the 400 bp fragment by using the primer pair VHH2-F and VHH2-R. The 400 bp fragment was purified and digested with *Sfi* I and *Not* I restriction enzymes (Takara), and ligated to pCANTAB 5E vector (Biovector Inc.). Therefore, phagemids for the phage display of VHHs were successfully constructed and were transformed into electro-competent *E. coli* TG1 cells. Then, 100 µL cells post-transformation were ten-fold serially diluted and spread on 2 × YTAG plates (containing 100 µg/mL ampicillin and 2% glucose), and incubated at 30 °C overnight to calculate the effective library size. The remaining cells post-transformation were spread on 2 × YTAG plates to incubate at 30 °C overnight.

### Identification of library diversity and capacity

A volume of 100 µL of the cultures, washed from plates after overnight incubation, was diluted in multiples of 10^− 1^, 10^− 2^, 10^− 3^, 10^− 4^, and 10^− 5^. Then, 100 µL of each dilution was spread onto LB/Amp plates and incubated overnight at 37 °C. Colonies were counted the next day, and 60 single colonies were randomly selected for positive rate identification by using Detc-F and Detc-R as primers. The plasmid was extracted from the positive colonies for sequencing and the library diversity was analyzed based on the sequencing results.

### Biopanning for phage libraries

Three rounds of panning were performed to enrich SRMV F and H protein-specific nanobodies. Briefly, one branch (1 mL) of the primary antibody library was inoculated into 200 mL 2×YT media, and incubated at 37 °C with shaking until OD_600nm_ up to about 0.6. Cells were infected with M13K07 helper phage (Moi = 20:1) at 37℃ for 30 min, and then centrifuged at 4000 rpm for 5 min to collect infected bacteria. 2 × YT media supplemented with 50 µg/mL kanamycin, 120 µg/mL ampicillin, and 1% glucose and incubated overnight at 37 °C with shaking at 200 rpm, and then centrifuged at 4000 rpm for 20 min. Phage was precipitated with 20% Polyethylene Glycol (PEG)/ 2.5 M NaCl in an ice bath at 4 °C for 1 h, and re-suspended in 5 mL of 2 × YT medium and the titer of the amplified phage library was determined.

Purified SRMV-F and -H protein was coated on the ELISA plate and incubated overnight at 4 °C. The plate was washed five times with PBS, and blocked for 2 h at 37 °C with 5% skimmed milk powder in PBS. Then the resuspended phage (4 × 10^11^ phage/mL) was added to a 96-well ELISA plate with 100 µL per well and incubated at 37 °C for 1 h. After the plate was washed five times, 100 µL of 0.2 M Gly-HCl buffer was added to each well for 10 min at room temperature to elute the retained phages, and neutralized with 30 µL of 1 M Tris-HCl (pH 7.5). The eluted phages were amplified in TG1 *E. coli* and rescued with M13K07 helper phage.

After four rounds of panning, infected cells were spread onto LB agar plates, and incubated at 30℃ overnight. 96 selected clones were then randomly selected from the plate and inoculated into two 96-well plates containing 100 µL 2 × YTAG medium, and incubated at 30 ℃ overnight. 2 µL of each cell suspension was transferred to two new 96-well plates containing 200 µL 2 × YTAG media, incubated at 37℃ with shaking at 140 rpm for 1.5 h. Then the plates were infected with M13K07 helper phage at 37℃ with shaking for 1 h, centrifuged at 3000 rpm for 25 min, and pellets were resuspended in 200 µL 2× YT medium containing 100 µg/mL ampicillin and 50 µg/ ml kanamycin. After plates were incubated at 30 ℃ with shaking overnight, the supernatants were subjected to ELISA for selection of strong binders.

### Phage-ELISA to detect nanobodies

The purified SRMV-F and -H proteins were respectively coated in 96-well ELISA plates at 1 µg/well and incubated overnight at 4 °C. The plates were washed thrice with PBS and blocked with 5% MPBS (200 µL per well). After discarding the blocking buffer, 100 µL amplified single phage clones were added to each well. After incubation at 37 °C and washing process, HRP-conjugate anti-M13 monoclonal antibody was added, and tetramethylbenzidine (TMB) was used for chromogenesis and incubating at 37 °C for 15 min and stopped by adding 50 µL 0.5 mol/L H_2_SO_4_. The absorbance was read using a microplate reader at 450 nm (Thermal). The clones that significantly reacted with F and H proteins (OD _450_≥1) were initially determined to be positive clones, which were subjected to sequencing after PCR identification and amino acid sequence analysis.

### Expression and purification of nanobody

Positive phage clones were used as template and the coding sequence of VHH fragments targeting SRMV structures proteins were amplified by PCR with the primer Exp-F and Exp-R and then cloned to the expression vector. Subsequently, the recombinant plasmid was transformed into *E. coli* BL21(DE3) cells and the recombinant cell was then induced by 0.5 mmol/L isopropyl-D-thiogalactopyranoside (IPTG) at 16℃ for 5 h. The bacterial pellet was collected by centrifugation and re-suspended with PBS and then sonicated for 20 min in four-second intervals. The supernatant and precipitate were collected to assess the expression of nanobodies by 12% sodium dodecyl sulfate polyacrylamide gel electrophoresis (SDS-PAGE). The nanobodies were further purified using the Ni-NTA Superflow Agarose column (Changzhou Smart-Lifesciences Biotechnology Co., Ltd.) according to the user manual.

### Western blotting

Recombinant VHH proteins were separated by 12% SDS-PAGE and transferred to the nitrocellulose (NC) membrane. NC membrane was blocked by 5% skimmed milk–PBS with 0.05% Tween 20 (PBST) at 37 °C for 1 h then washed twice with TBST (Tris-buffered saline containing 0.5% Tween 20). The purified nanobodies (1 mg/mL) were applied at 1:1000 dilution with 1-hour incubation at 37 °C, after five washes with wash buffer (0.05% Tween 20 in PBS), followed by 1 h of incubation at room temperature with mouse anti-flag antibodies and then the membrane was incubated for 1 h in HRP-conjugated goat anti-mouse IgG (1:5,000) followed by washing. The protein bands were visualized using a chemiluminescent substrate (SuperSignal West Dura Extended Duration Substrate, Thermo-Fisher, Rockford, IL, USA) according to the manufacturer’s instructions. The image was captured and analyzed using a ChemiImager™ 4,400 Low Light Imaging system (Alpha Innotech Corporation, San Leandro, CA, USA).

### ELISA

ELISA was performed to detect the binding activity between SRMV and nanobodies prepared in this study. In brief, inactivated SRMV particles were coated onto 96-well ELISA plates at 1 µg/well and incubated overnight at 4 °C. The plates were washed five times with TBST. The purified nanobodies (1 mg/mL) were diluted 10-fold and added to the wells, followed by incubation at 37 °C for 1 h. After washing with wash buffer (0.05% Tween 20 in PBS), mouse anti-flag antibodies were added to the wells, followed by HRP-conjugated goat anti-mouse IgG sequentially. The reaction was developed by adding 100 µL tetramethylbenzidine (TMB), incubating at 37 °C for 15 min, and stopped by adding 50 µL 0.5 mol/L H_2_SO_4_. The absorbance was read using a microplate reader at 450 nm (Thermal).

### Virus neutralization test (VNT)

A virus Neutralization Test (VNT) was performed as described by Rossiter et al. [32]. to determine the neutralizing nanobody titer using Vero cells. Briefly, 100 TCID_50_/100 µL of SRMV were incubated with 100 µL of 2-fold serially diluted nanobody samples in a 96-well tissue culture plate for 1 h at 37 °C in a 5% CO_2_ incubator. Then, 100 µL of Vero cell suspension (10^6^ cells/mL) was added to each well and incubated again at 37 °C for 6–7 days, wells were examined for cytopathic effect (CPE) daily under an inverted microscope and the final reading was taken on the 7th day post-inoculation. Neutralizing nanobody titers were calculated as the reciprocal of the highest dilution of nanobody that shows no CPE in 50% of wells using the Reed-Muench method. The VN titers > 8 were considered as protective. In each plate, the following controls were set up: Vero cell control (CC) with only cells, nanobody toxicity control group, positive and negative sera controls, and virus control (VC) with virus and cells.

## Results

### Construction of an immune library and bio-panning

**Fig. 1:**
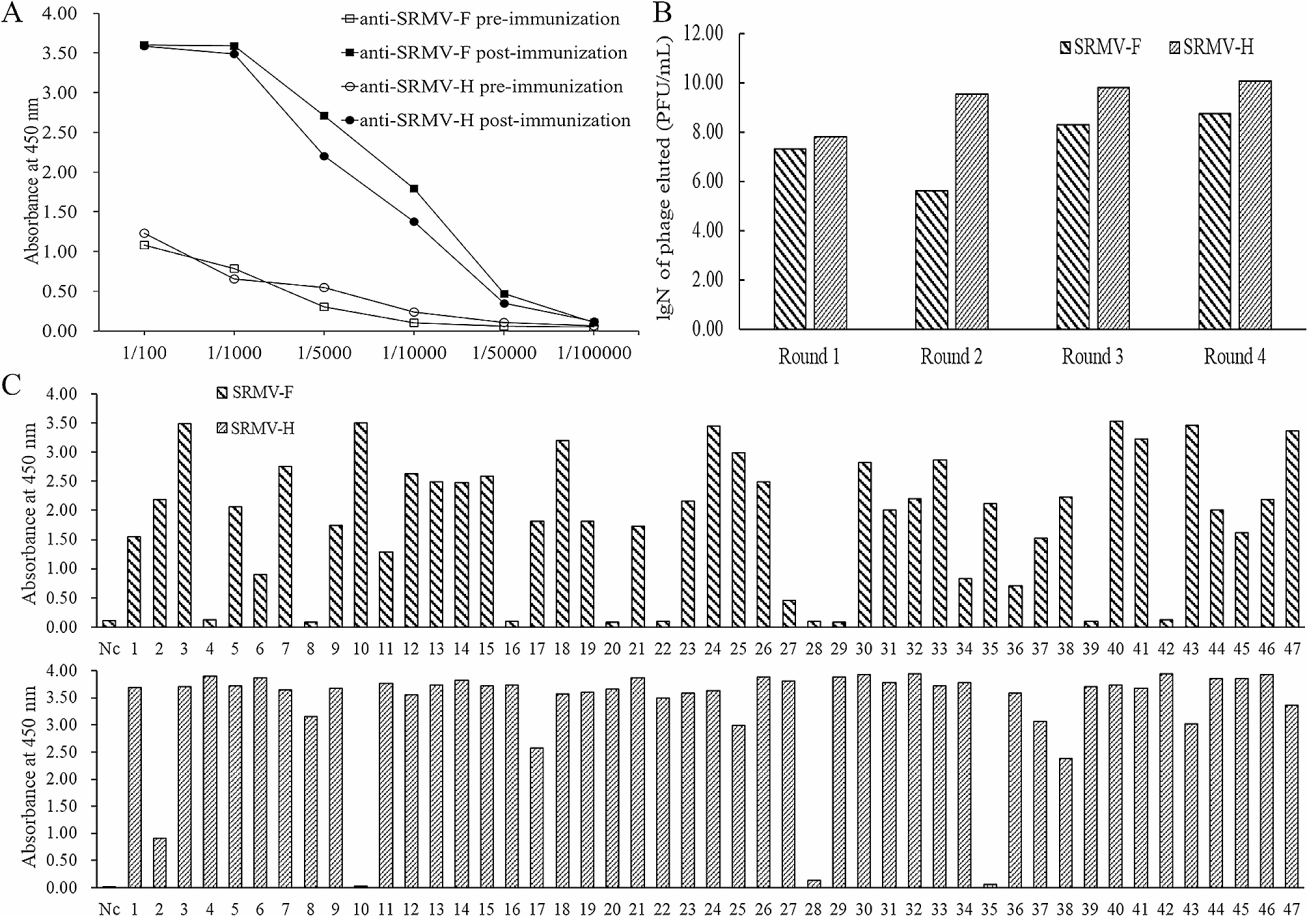
Serum antibody titer after immunization and biopanning process monitoring. **(A)** Serum antibody titer against SRMV-F and –H after immunization procedure, which were both up to 1:50000. **(B)** Enrichment evaluation of phage-displayed nanobodies by biopanning. Phage-displayed nanobodies were selected against SRMV-F and –H protein respectively, and *E. coli* TG1 was infected with bound phage. The number of phages eluted after each round of biopanning was counted based on the number of plaques (PFU/mL) formed after infection of the host bacteria with the eluted phage particles. **(C)** ELISA assay for selection of individual phage particles against SRMV-F and -H protein, respectively

In order to construct an immune phage-displayed library from the VHH repertoire of alpaca, a male adult alpaca was immunized six times with SRMV-F and -H protein, and the immune efficacy was analyzed using ELISA. As shown in Fig. 1A, the titer of SRMV-F and -H specific antibody in alpaca serum was up to 1:5 × 10^4^ after immunization. VHH coding gene fragment was obtained by two-step nest PCR procedure after generating cDNA based on the total mRNA that was isolated from PBMCs, and the VHH gene fragments, after purification were cloned into phagemid vector pCANTAB 5E and transfected into *E. coli* TG1. The size of this VHH library was 2.26 × 10^10^ CFU/mL with a positive rate of 96.4%.

Phage particles displaying nanobodies were rescued by M13K07 helper phage and were panned using SRMV-F and -H protein coated in 96-microplates wells, respectively. The enrichment of phage-displayed nanobodies of each round of biopanning was monitored by titrating phage eluted from positive wells. Results showed that phage-displayed nanobodies against SRMV-F and -H proteins were both enriched respectively after four rounds of biopanning, and the number of enriched phage particles binding to SRMV-F and -H obtained from the fourth round of panning was 28 and 186.9 times more than that obtained from the first round of panning, respectively (Fig. 1B). Comparing the number of phage particles against SRMV-F and -H eluted from each round of biopanning, the enrichment efficacy of SRMV-H specific phage-displayed nanobodies was better than that of SRMV-F specific phage particles (Fig. 1B). In general, it is sufficient to identify high affinity SRMV specific nanobodies from the four-rounds enriched SRMV-F and -H specific phage reservoir.

**Fig. 2:**
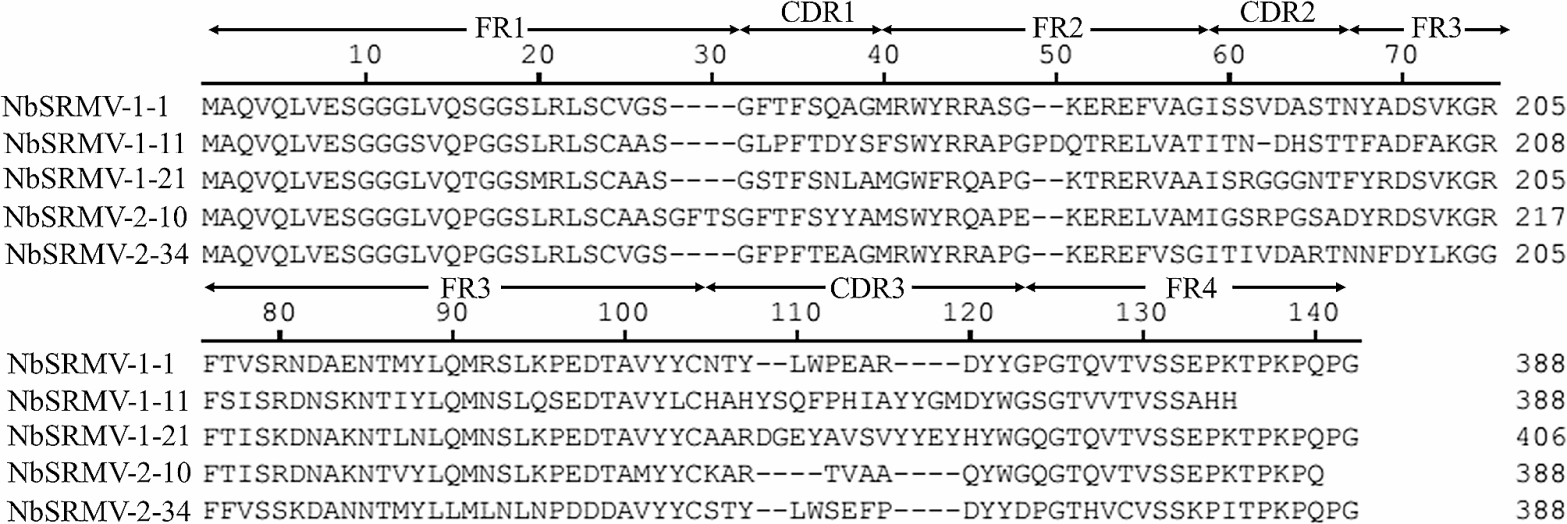
Amino acid sequence of anti-SRMV nanobodies after four rounds of biopanning. The frameworks and complementary determining regions were determined according to IMGT

After four rounds of biopanning, forty-seven phage particles obtained from SRMV-F coated wells and SRMV-H coated wells were randomly selected, respectively, and were evaluated by ELISA. Thirty-eight clones obtained from SRMV-F coated wells showed positive and forty-four phage particles from SRMV-H coated wells were positive (Fig. 1C). And these positive clones were selected and sequenced. As shown in Fig. 2, there were total five independent different sequences were identified, namely, NbSRMV-1-1, NbSRMV-1-11, NbSRMV-1-21, NbSRMV-2-10, and NbSRMV-2-34, three of which (NbSRMV-1-1, NbSRMV-1-11, NbSRMV-2-34) were obtained from SRMV-F coated wells and the other two clones were obtained from SRMV-H coated wells.

### Expression, purification and identification of anti-SRMV nanobody

**Fig. 3:**
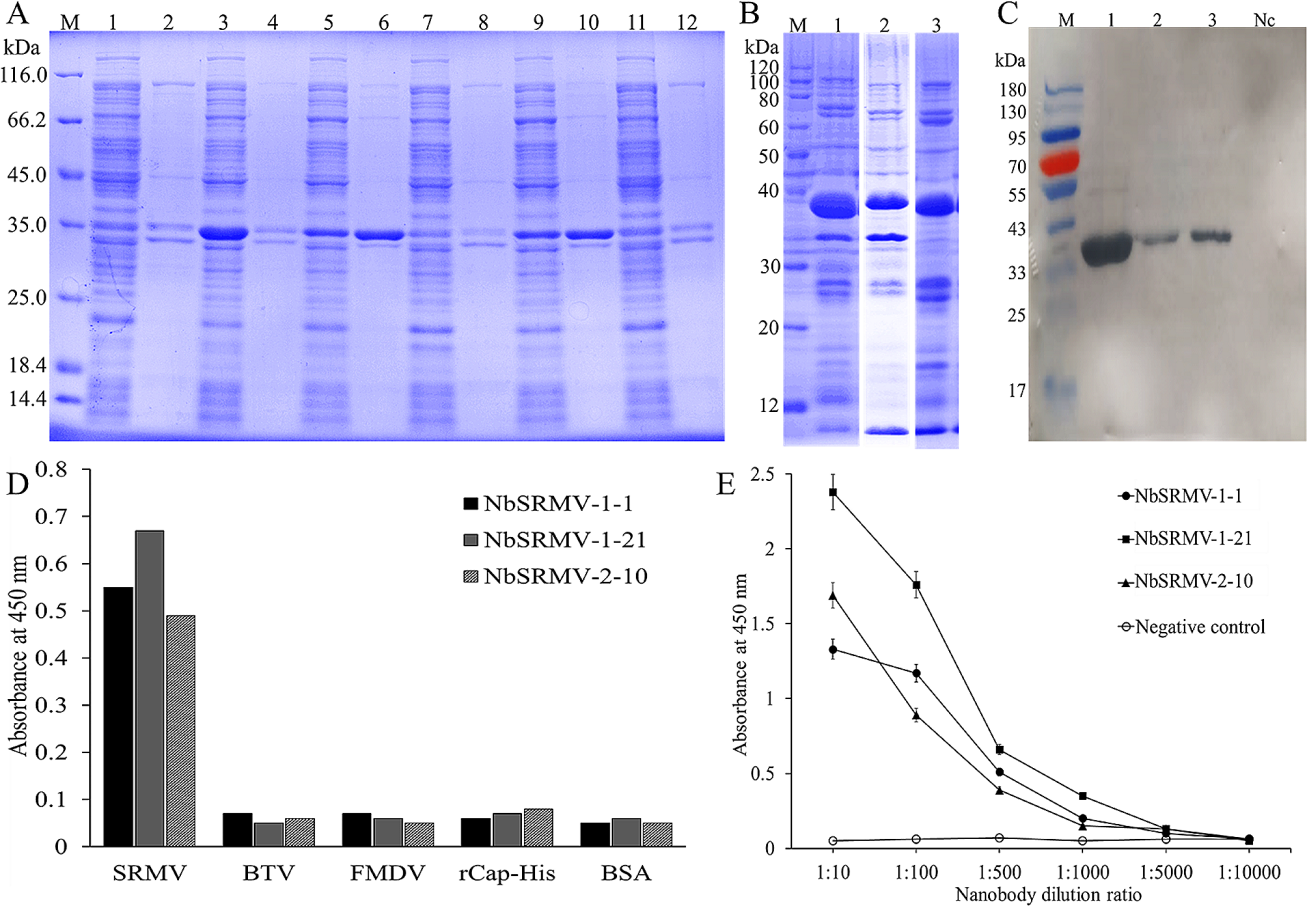
Expression and identification of nanobodies against SRMV. **(A)** Prokaryotic expression of nanobodies against SRMV (M standard protein marker, 1–2 supernatant and precipitation of negative bacteria respectively, 3–4 supernatant and precipitation of NbSRMV-1-1 expression bacteria respectively, 5–6 supernatant and precipitation of NbSRMV-2-10 expression bacteria respectively, 7–8 supernatant and precipitation of NbSRMV-1-11 expression bacteria respectively, 9–10 supernatant and precipitation of NbSRMV-1-21 expression bacteria respectively, 11–12 supernatant and precipitation of NbSRMV-2-34 expression bacteria respectively). **(B)** Purification of nanobodies against SRMV (M standard protein marker, 1 purified NbSRMV-1-1, 2 purified NbSRMV-2-10, 3 purified NbSRMV-1-21). **(C)** Western blotting analysis of nanobodies against SRMV (M standard protein marker, 1 NbSRMV-1-1, 2 NbSRMV-2-10, 3 NbSRMV-1-21, Nc BSA negative control). **(D)** Specificity analysis of nanobodies against inactivated SRMV. **(E)** Binding affinity analysis of the nanobodies against inactivated SRMV using ELISA

The DNA fragments of NbSRMV-1-1, NbSRMV-1-11, NbSRMV-1-21, NbSRMV-2-10, and NbSRMV-2-34 were cloned into the prokaryotic expression vector pcold-sumo and were then transformed into *E. coli* BL21(DE3), respectively. The nanobodies carrying C-terminal 6×His-tag were expressed and purified by Ni-NTA affinity columns. As shown in Fig. 3A-C, the band of the recombinant nanobodies in SDS-PAGE gel was about 35 kDa, which met the theoretical values in size. NbSRMV-1-1 was expressed in soluble form, with NbSRMV-2-10 and NbSRMV-1-21 partially soluble (Fig. 3A). However, NbSRMV-1-11 and NbSRMV-2-34 failed to express (Fig. 3A). The recombinant nanobody proteins, NbSRMV-1-1, NbSRMV-2-10, and NbSRMV-1-21 were identified using western blotting (Fig. 3C).

### Specificity and affinity analysis

The specificity of nanobodies was evaluated using ELISA whereby the binding ability to inactivated SRMV was compared to inactivated bluetongue virus (BTV), inactivated foot-and-mouth disease virus (FMDV), 6×his-tag labeled porcine circovirus type 2 recombinant Cap protein (rCap-His), and Bovine serum albumin (BSA). Results indicated that NbSRMV-1-1, NbSRMV-2-10, and NbSRMV-1-21 showed good specificity towards inactivated SRMV, and no cross-reactivity with other antigens was observed (Fig. 3D).

In order to characterize the affinity of the three nanobodies by ELISA, serials of dilution of these nanobodies were used to react with inactivated SRMV coated in 96-microplates wells. As shown in Fig. 3E, the ELISA titer of the three nanobodies towards inactivated SRMV was up to 1:1000, and NbSRMV-1-21 displayed the highest affinity towards inactivated SRMV (Fig. 3E).

### Virus neutralization test

The virus-neutralizing potency of NbSRMV-1-1, NbSRMV-2-10, and NbSRMV-1-21 was evaluated using Vero cells according to the cytopathic effect (CPE). The CPE results demonstrated that NbSRMV-1-1 and NbSRMV-2-10 failed to neutralize SRMV resulting in CPE of Vero cells, and NbSRMV-1-21 showed neutralization to SRMV with the maximum dilution of 1:4 (0.25 µg/mL). The neutralization ability of the nanobody was concentration-dependent.

## Discussion

SRMV affects mainly small domestic ruminants (sheep and goats) and camels resulting in serious economic losses, especially in many countries of Africa and Asia [1, 24]. Growing evidences suggest that the SRMV host range has expanded to large ruminants and wildlife animals, such as gazelles, deer, roe deer, and antelope [25–31]. The potential role of wildlife species as maintenance hosts for SRMV in these different ecosystems is unknown [32], contributing to virus persistence in multi-host systems with an impact on PPR control and eradication program [1, 33].

Diagnostic tools for SRMV detection are primarily developed for livestock species [32]. The laboratory diagnosis of PPR disease is mainly based on virus isolation, but it requires laboratories with tissue culture facilities and is less sensitive [34]. The available diagnostic methods for immunological/serological assays, include ELISA, VNT, PPR-luciferase immunoprecipitation systems, and pseudo type-based neutralization assays [35–37], and they have both value and shortcomings. The vaccines currently used in the field against SRMV are live attenuated vaccines [38], for example, two live attenuated vaccines (Nigeria/75/1and Sungri/96) are available for the control of PPR with a reasonable success rate, but serological tests cannot distinguish naturally infected and vaccinated animals [39]. In addition, in most countries where SRMV is endemic, the maintenance of cold storage and transport facilities can be problematic and could lead to vaccine administration in poor immunization conditions [38]. Clear guidelines and standards for application and interpretation of PPR diagnostic tests in wildlife species need to be established, and rapid, accurate and cost-effective diagnostic methods and therapeutic antibodies are required for SRMV.

The VHHs found in camels, llamas, and sharks [11] have emerged as a new hope as they possess the unique properties of small molecular size (15 kDa), low immunogenicity, strong tissue penetrating ability, high binding affinity and stability [40]. Multiple reports suggest that dromedaries are susceptible to SRMV, as observed in Ethiopia, Sudan and Iran [41–46]. Serological surveys have demonstrated that camels are susceptible to infection and in some instances may express a severe illness (respiratory distress) and mortality [41, 43]. Therefore, the immunized alpacas provide direct access to the in vivo affinity-matured antibodies. In 2021, Kinimi et al. [47] immunized alpacas with a live attenuated vaccine strain (SRMV/N/75/1), and nine nanobodies that specifically recognize completely inactivated SRMV were identified using ELISA showing superb potency in rapidly identifying SRMV. This study confirms the practicality of isolating a panel of SRMV-specific nanobodies from an immunized alpaca without having prior knowledge of the antigens involved. Liu et al. [48] immunized camels with the SRMV vaccine and four SRMV VHHs were selected and characterized, which were shown to have biological activity, close affinity to SRMV, and no cross-reaction with other sheep disease antigens. These nanobodies against SRMV antigens may mark the beginning of the use of nanobodies as analytical tools for the diagnostic and therapeutic of SRMV infection in the future.

The F and H proteins of SRMV play crucial roles in the interaction between the virus and cells. H protein is involved in target-cell attachment and generally regulates viral adsorption and entry, determining pathogenicity, and releasing newly-produced viral particles [49–51], while the F protein is believed to disrupt the target cell membrane leading to the virus–cell and cell–cell fusions [52, 53]. Given the significance of the F and H proteins of SRMV, we successfully generated an immune VHHs library by using SRMV-F and -H proteins, and three nanobodies against SRMV were expressed using a prokaryotic expression system. To assess the binding specificity of three nanobodies, inactivated SRMV was used for the assessment in an ELISA assay. Our results preliminarily showed that three of the selected nanobodies displayed excellent specificity. VNTs are often considered as the gold standard for serological detection, as the results demonstrate the ability to neutralize infectious viruses. In our study, NbSRMV-1-21 displayed neutralization towards SRMV at a maximum dilution ratio of 1:4. In addition, our data illustrate the potential usefulness of applying ELISA and VNT antibody tests in parallel to enhance the sensitivity of infection detection, as both ELISA and VNT tests have positive credentials to bring to diagnostics.

In conclusion, we generated SRMV-reactive nanobodies that showed superb potency in rapidly identifying SRMV, which provides a new, promising reagent tool for the development of SRMV-based nanobody drugs and diagnostic kits.
